# Remote endarterectomy to remove infected Viabahn stent-graft

**DOI:** 10.1016/j.jvscit.2021.04.020

**Published:** 2021-05-20

**Authors:** Christopher L. Tarola, Morgan Young-Speirs, John W.D. Speirs, Carman M. Iannicello

**Affiliations:** a Division of Cardiac Surgery, Department of Surgery, University Hospital, London Health Sciences Center, London, Ontario; b Cumming School of Medicine, University of Calgary, Calgary, Alberta; c Department of Diagnostic Imaging, Ouellette Campus, Windsor Regional Hospital, Windsor, Ontario, Canada; d Division of Vascular Surgery, Department of Surgery, Ouellette Campus, Windsor Regional Hospital, Windsor, Ontario, Canada

**Keywords:** Vascular surgery, Stent-graft removal, Endarterectomy, Case study

## Abstract

Infection of peripheral arterial vascular grafts and stent-grafts represents a complex surgical scenario, with a number of proposed management strategies. Surgical removal of infected material with adjunctive arterial reconstruction is often required. However, surgical removal is often difficult and complex. This case study demonstrates an infected Viabahn stent-graft between the external iliac artery and the superficial femoral artery, with arterial autolysis of the common femoral artery and proximal superficial femoral artery, in which a hybrid technique combining remote endarterectomy and surgical debridement was used to remove the infected stent-graft.

Prosthetic graft infection after vascular bypass is a challenging surgical complication, especially when compounded by acute hemorrhage. Clinical experience dictates that radical excision of infected material and extra-anatomical bypass in a noninfected field is the treatment of choice.^1^, ^2^, ^3^, ^4^ Several other management strategies have been proposed, including in situ vein reconstruction, antibiotic-impregnated grafts, negative pressure wound therapy, and simple ligation.^1^, ^2^, ^3^, ^4^ Less extensively investigated are incidents of prosthetic endovascular stent-graft infection, despite their use in managing infected native vessels and grafts.^1^^,^^5^^,^^6^ Utilization of endovascular strategies is attractive given the associated reduction in surgical morbidity and mortality, especially in high-surgical-risk patients. Although there are limited reports describing management of stent-graft infections, particularly in infrainguinal vessels, the true incidence is likely underestimated because of under-reporting and appreciation by treating physicians. This case study demonstrates a novel, hybrid strategy to surgical explantation of an infected stent-graft, using remote endarterectomy to reduce surgical intervention. The patient consented to publication of the details and images of this case.

## Case report

A 71-year-old woman, with a history of hypertension, dyslipidemia, hypothyroidism, and morbid obesity (body mass index 40), initially presented with one-block calf claudication and nocturnal rest pain of the right leg. Angiography demonstrated heavy calcification within the right external iliac (EIA), common femoral (CFA), and profunda arteries (PFA), and occlusion from the origin of the superficial femoral artery (SFA) to popliteal artery. A femoropopliteal in situ bypass with iliofemoral endarterectomy, extended profundoplasty, and bovine pericardial patch (LeMaitre, Burlington, Mass) arterioplasty was performed. The saphenous vein graft originated from the bovine patch. She was discharged on oral antibiotics given her high risk for wound infection.

She presented 1 month later with infection and bleeding in the right inguinal incision. Angiography demonstrated a patent graft, and computed tomography suggested an infected hematoma. This was initially treated with intravenous (IV) antibiotics. However, secondary to hemorrhage from the groin, she underwent emergency excision of the infected pseudoaneurysm and bovine patch via a right flank retroperitoneal incision to control the inflow external iliac artery; she then underwent vein patch arterioplasty of the CFA and PFA, excision of the proximal portion of the previous femoropopliteal bypass due to sepsis, and reconstruction of the right femoropopliteal bypass using the left long saphenous vein. Because of extensive sepsis and debridement, a full thickness skin, fat, and muscle flap was rotated from the anterior thigh to the right groin. The patient bled postoperatively, necessitating stenting grafting of the right CFA into the right PFA using a 10 mm × 100 mm Viabahn (W. L. Gore and Associates, Inc, Flagstaff, Ariz) stent graft, which occluded her femoral-to-popliteal bypass.

**Fig 1:**
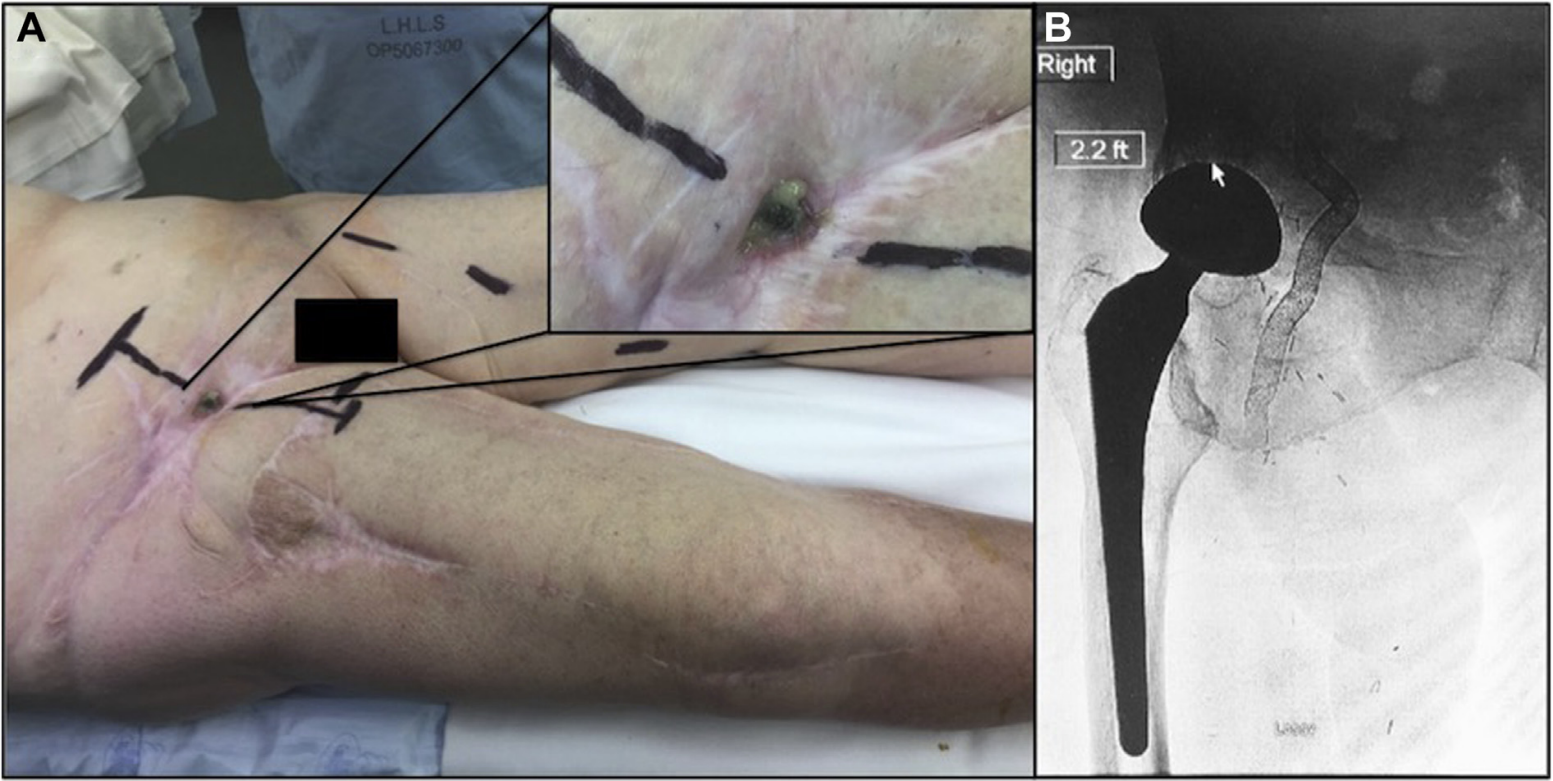
**A,** Exposed, infected right common femoral Viabahn stent graft visible at the skin. **B,** Preoperative angiography demonstrating the location of the Viabahn stent graft extending from the right external iliac artery to the right common femoral artery.

She was discharged on IV antibiotics but re-presented 5 months later with a history of a previous hemorrhage from the groin that had stopped. She was initially treated for methicillin-resistant *Staphylococcus aureus*. The patient was reluctant to undergo further surgery. After negative blood cultures, a 10 mm × 150 mm Viabahn graft was deployed extending from the EIA into the previously placed graft, thereby excluding a large false aneurysm proximal to the original stent graft. She was discharged and remained on a continuous 2-year course of IV antibiotics due to a chronically draining sinus in the right groin, and positive *Escherichia coli, Enterobacter cloacae,* methicillin-resistant *S. aureus*, and *Klebsiella pneumonia* cultures. Despite ongoing antibiotics, eventually the Viabahn graft eroded the overlying skin and was visible in the right groin (Fig 1).

**Fig 2:**
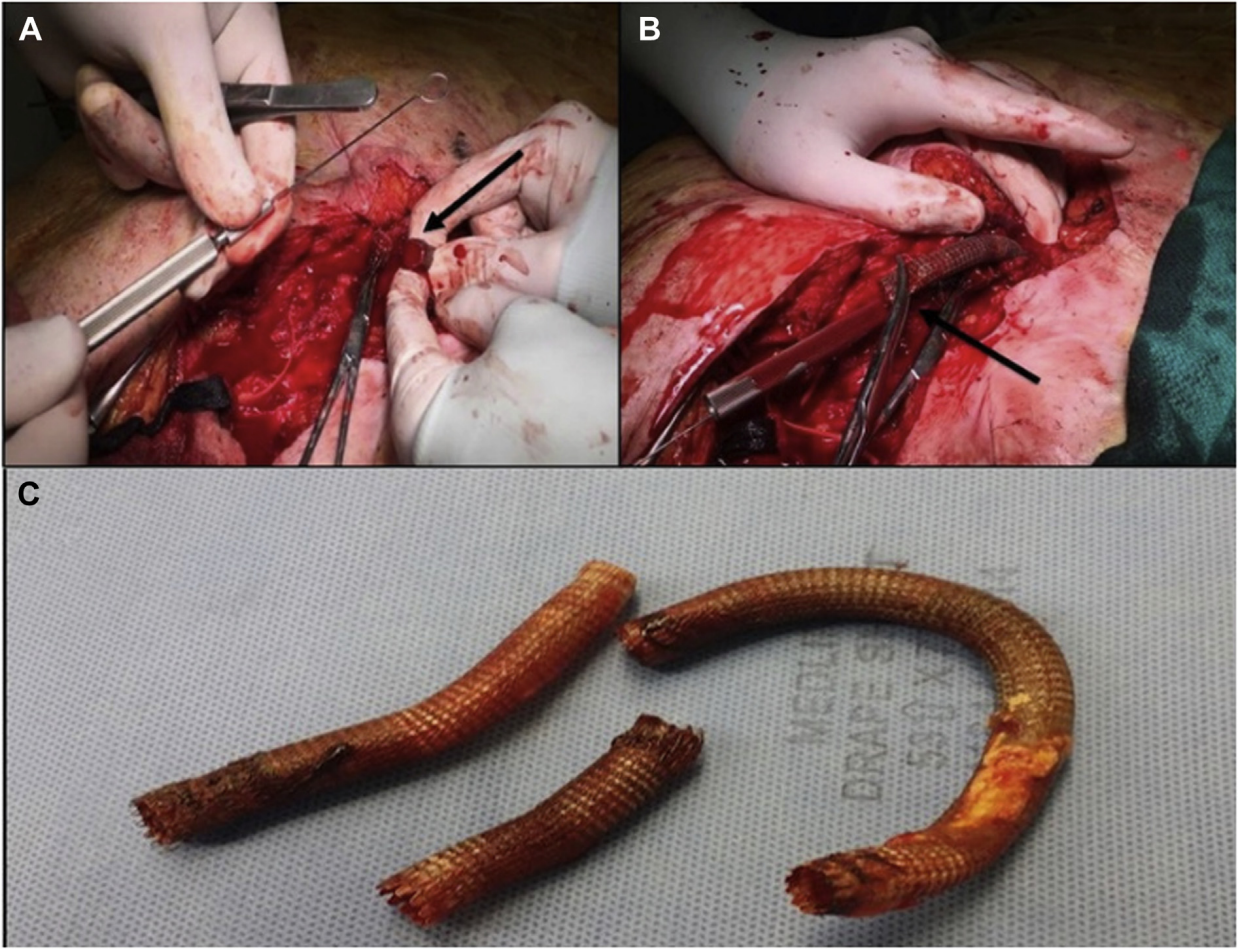
**A,** Isolation of the distal end of the right Viabahn stent graft (*arrow*) from the right common femoral artery before the placement of the remote endarterectomy device. **B,** Remote endarterectomy device placed with a loop surrounding the distal end of the stent graft (*arrow*) as “endarterectomy core.” **C,** Explanted Viabahn stent-grafts.

**Fig 3:**
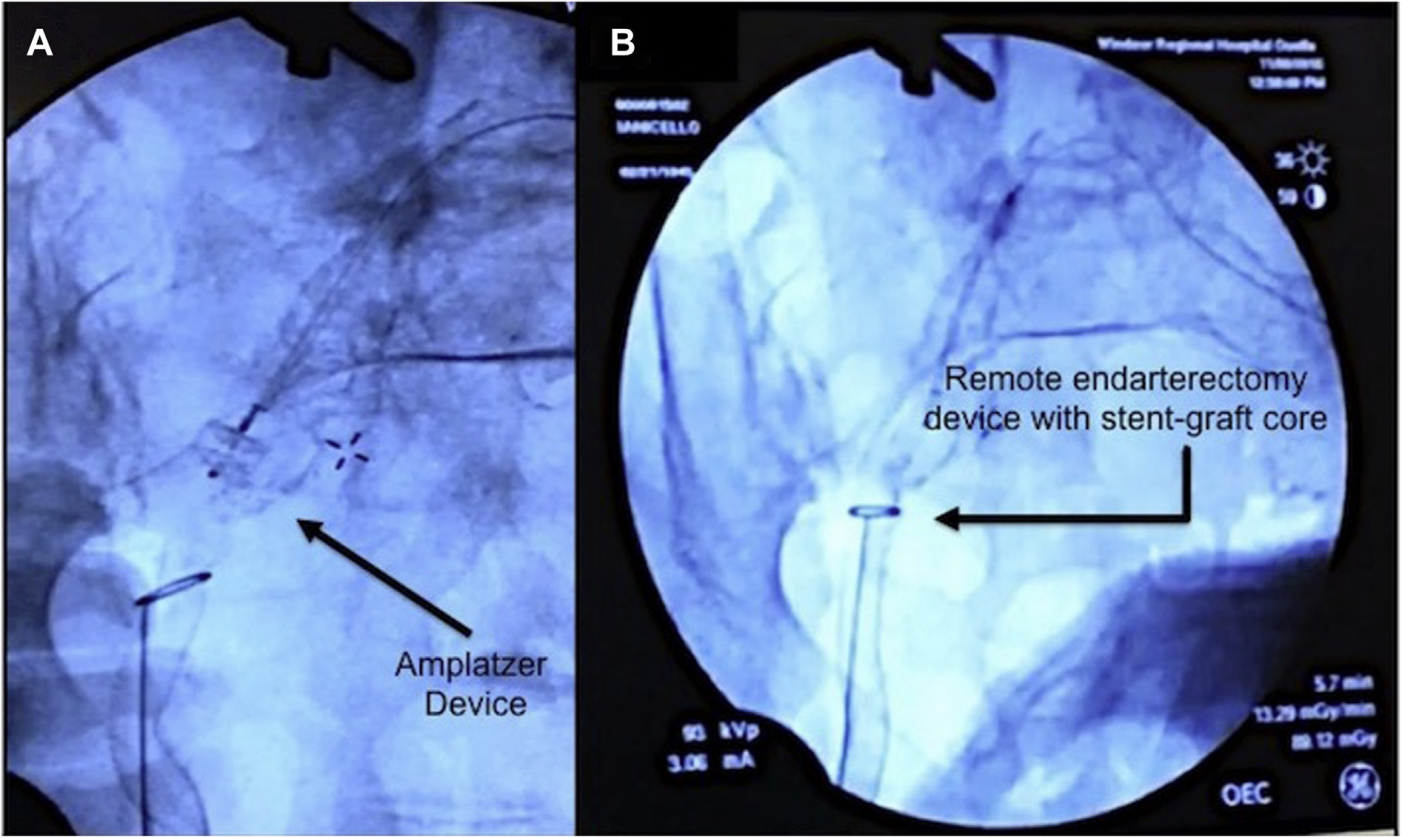
**A,** Deployment of Amplatzer. **B,** Remote endarterectomy device surrounding the Viabahn stent-graft core.

She was subsequently taken to the operating room for definitive removal of the infected stent-graft. The patient was placed on a Jackson table to permit intraoperative use of angiography, using a mobile digital C-arm angiographic system (GE Healthcare, Buckinghamshire, United Kingdom). Using a vertical incision from the lower anterior abdominal wall to the right thigh, the PFA (at the level of the second perforating muscular branch) and proximal CFA were exposed, and vascular control was obtained. The CFA and proximal PFA were autolyzed by sepsis, and only the stent-graft remained (Fig 2). Simultaneously, the left femoral vein was harvested. At this point with the patient systemically heparinized, a 5-Fr Racket catheter was inserted into the left CFA. A 12 mm Amplatzer (St. Jude Medical, St. Paul, Minn) vascular plug was placed at the proximal end of the previously placed stent graft using a 6-Fr Terumo guide catheter into the proximal right EIA (Fig 3).

After confirming occlusion of the right EIA via angiography, the distal profunda femoris was clamped and the stent graft was transected. Using sharp dissection, the distal portion of the stent graft was excised to the mid-profunda. Attention was then directed proximally. Direct moderate traction on the transected end of the stent-graft did not dislodge it. An EndoRE remote endarterectomy device (LeMaitre Vascular) was placed around the exposed graft, similar to an intimal core during remote endarterectomy (Figs 2 and 3). The device was gradually passed proximally under fluoroscopic guidance using a gentle, rotating technique, until the proximal end of the Viabahn graft was reached, resulting in separation of the graft from the remaining artery. The Viabahn graft was removed entirely (Fig 2). The proximal CFA was oversewn, and a left CFA to right PFA ex situ bypass using the harvested femoral vein was performed. The wounds were closed primarily.

Postoperatively, the patient had minor wound sepsis. The patient has been followed yearly for 4.5 years with duplex scanning of the patent cross-femoral venous bypass. She has had full wound healing and remains asymptomatic.

## Discussion

Although uncommon, peripheral extra-anatomical graft and endovascular stent-graft infection can create complex surgical scenarios with significant patient morbidity. Incidence of infected peripheral bare-metal stents is estimated at 1 in 10,000,^7^ with scattered cases reported.^7^, ^8^, ^9^ Infection associated with stent-grafts, namely Viabahn, seems equally rare given limited reporting in literature. A literature review identified three reports describing stent-graft incision—two involved Hemobahns, and one involved a Viabahn graft, each deployed in the SFA.^10^, ^11^, ^12^ This report not only contributes to this literature but also emphasizes the importance of aggressive surgical intervention to remove infected material, arterial debridement to healthy margins, and arterial reconstruction with autologous conduit. In spite of various reports demonstrating eradication of infection without stent excision,^7^^,^^13^ in this patient, infection and arterial autolysis persisted despite years of antibiotic therapy.

Arterial infection can cause arterial destruction in the absence of foreign material; however, the presence of foreign material further predisposes ongoing arterial suppuration and degradation.^7^^,^^8^^,^^10^^,^^14^^,^^15^ In this case, after infection of the bovine patch, all foreign material was replaced. The saphenous vein was used to replace the bovine patch and proximal portion of the bypass. The reconstruction was then covered with a muscle flap.^16^, ^17^, ^18^ However, because of significant bleeding postoperatively, a Viabahn stent was placed given the patient's high surgical risk. Importantly, a portion of the morbidity and mortality associated with graft infection should be ascribed to the surgical complexity of removing infected foreign material within intra-abdominal and thoracic arteries, substantiating the decision to use remote endarterectomy to eliminate the need for retroperitoneal or abdominal incision.

Remote endarterectomy has been shown to be safe for the management of peripheral arterial disease, though with variable results.^19^, ^20^, ^21^ An Amplatzer plug was used to occlude the iliac artery distal to the bifurcation of the EIA and internal iliac artery, thereby reducing the risk of bleeding after remote endarterectomy and avoiding occlusion of the internal iliac artery.

Given the formidable operative morbidity and mortality associated with stent infections, a hybrid surgical technique was employed in this case. Accordingly, retroperitoneal exposure was avoided, fluoroscopy-guided remote endarterectomy reduced surgical invasiveness in a high-risk patient, and removal of infected prosthetic material promoted wound healing. The treatment principles for management of infected covered stents in peripheral arteries mirror those of infected aortic endografts of the abdominal aorta—surgical explantation of the infected graft.
